# From End Treatment to Source Prevention: Socio-Ecological Approaches to Promote Research on the Environment and Non-Communicable Chronic Diseases with Special Reference to China

**DOI:** 10.3390/ijerph16111900

**Published:** 2019-05-29

**Authors:** Xi-Zhang Shan, Yong Li, Kun Lai

**Affiliations:** 1 School of Geographical Sciences, South China Normal University, 55 Zhongshan Avenue West, Tianhe District, Guangzhou 510631, Guangdong, China; 2 Key Lab of Guangdong Province for Utilization of Remote Sensing and Geographical Information System, Guangzhou Institute of Geography, Guangzhou 510070, Guangdong, China; liyong@gdas.ac.cn; 3 School of Tourism Management, Sun Yat-sen University (Zhuhai Campus), Tangjiawan Town, Zhuhai 519082, Guangdong, China

**Keywords:** environmental intervention, comprehensive research framework, integrative research approaches, NCD prevention, socio-ecological model

## Abstract

Globally, the pandemic of non-communicable chronic diseases (NCDs) has become a critical public health problem. Although NCD prevention has been shifting from individual behavioral interventions to broad environmental interventions, it is still necessary to promote research on the environment and NCDs as a whole. Therefore, this conceptual paper aimed to develop a general and novel framework to advance this line of research. The framework uses socio-ecological approaches that emphasize source prevention rather than the end treatment. Specifically, this framework comprehensively covered integrative research approaches, prioritized areas, urgent efforts, innovative methodologies, and improved funding. The framework used China as a typical context, where its public health policies, similar to other nations, still focus on the end treatment of NCDs, placing emphasis on biomedical approaches and technologies. China’s relevant efforts may furnish new insights and approaches concerning NCD prevention, and these efforts may benefit the improvement of global health and well-being. Such social-ecological research efforts can help to accelerate a shift from existing individual interventions to environmental interventions, thereby ultimately achieving the effective source prevention of NCDs in China and around the globe.

## 1. Introduction

Non-communicable chronic diseases (NCDs), especially cardiovascular diseases, cancers, chronic respiratory diseases, and diabetes, have aroused increased global concern. NCDs are responsible for 63% of deaths globally, which mostly occur in low- and middle-income countries [1]. Meanwhile, in China, NCDs have become a significant public health problem, where one in five Chinese have a kind of NCD. This accounts for 86.6% of all deaths in the country, where cardio-cerebrovascular diseases, cancers, and chronic respiratory diseases are the key causes. Moreover, the number of cases of chronic diseases, including hypertension, diabetes, and cancers, increased from 2002 to 2012 [2,3]. The incidences of overweight and obesity, which are associated with the development of multiple NCDs, have significantly increased amongst Chinese adults (at 30% and 12%, respectively) over the past ten years [3]. Currently, China is experiencing an epidemiological transition (from communicable diseases) to NCDs [4]. The continued urbanization and aging taking place in the country have further accelerated this transition [5,6]. Consequently, the substantial increases in treatment costs, concomitant decreases in workforce productivity, and the resultant poverty will engender serious socioeconomic problems [7].

However, China’s health care system, which traditionally focuses on acute and infectious diseases, has remained unprepared to tackle the upsurge of NCDs. Thus far, the limited response by the system has been largely targeted towards setting up relevant policies, management, and technical strategies, as indicated in the two National Plans for the Prevention and Treatment of Chronic Diseases in China (2012–2015; 2017–2025) [2,8]. Evidence-based information on NCDs remains insufficient. Therefore, China needs stronger studies on the formulation of sound NCD policies and effective prevention strategies. As a result, this study aims to provide a general framework based on socio-ecological approaches to promote research on the source environment and NCDs, with special reference to China. The study will further provide evidence for NCD prevention by promoting a shift from current individual behavioral interventions to broad environmental interventions, thereby ultimately achieving effective source prevention.

## 2. Global Shift in NCD Prevention from Individual Behavioral Interventions Towards Broad Environmental Interventions Based on Socio-Ecological Approaches

NCD prevention has encountered numerous challenges despite the emergence of effective strategies [9]. NCDs are inherently lifestyle-related diseases caused by unhealthy eating, smoking, physical inactivity, etc. [1]. However, interventions that focus on individual behavior and lifestyle are considered ineffective or associated with limited success in terms of behavioral changes [10,11,12]. For example, lifestyle interventions elicit limited effects on the sedentary behavior of adults at risk [13]. Scholars have focused on the upstream factors contributing to NCDs, such as social, built environments (e.g., residential density, land use mix, and street connectivity), and policies, as well as broad approaches beyond individual behavior [10,14,15,16].

Genetics research has demonstrated modest effects of genetics on human health, particularly NCDs [17,18], and attention has been drawn to environmental factors or gene–environment interactions [14,19,20]. For example, Wu et al. (2016) used four independent approaches and estimated that extrinsic or environmental factors account for 70%–90% of the most common cancers [21]. Zhu et al. (2016) indicated that the intrinsic factors (stem cell mutagenesis) and extrinsic factors that promote the proliferation of stem cells jointly determine cancer risk, based on direct experimental evidence across multiple organs [22]. The increase in the obesity pandemic relates to the obesogenic environment rather than to genetic mutations [23,24]. Social factors may strongly affect cognitive decline amongst older people, and they may reduce the small influence of genotypes on cognitive functions [14]. Some social network features are linked to the incidence of obesity [25]. Furthermore, the effect of the built environment on active travel, depression, and diabetes has been reported [26,27,28,29,30]. Meanwhile, the greenness around residences has been shown to be linked to cognitive decline [31].

As such, interventions on individual behaviors and lifestyles are insufficient to address the increasing incidence of NCDs; thus, the characteristics of the environment need investigation. The effective prevention of NCDs necessitates a shift from individual behavioral interventions to environmental and policy interventions.

Increasingly, the socio-ecological or environmental approach to NCDs [23,32] is gaining use in environmental interventions research. Technical advances, such as big data and multilevel analysis [33], have facilitated this research. Public health scholars have established socio-ecological models to investigate physical activity, cardiovascular diseases, diabetes, and environmental interventions [15,16,34]. For example, an EU project in Madrid explored the four urban environmental domains of tobacco, physical activity, alcohol, and food environments, all of which are considered to be directly associated with individual NCD risk factors [35]. This approach transcends individual lifestyle and emphasizes environmental influences, which typically operate at multiple levels and time scales with complex interactions [14,16,33,36]. It also provides an integrative conceptual framework for NCD research and prevention. Moreover, this approach has been verified using empirical evidence, where examples include the effectiveness of promoting the cycling of integrative interventions based on socio-ecological models [37].

## 3. NCD Prevention, Research, and Funding in China

Prevention is the principal national strategy to address NCDs in China [6,8]. However, feasible and effective interventions remain uncertain, and efforts have been considerably restricted to specific projects and short-term fragmented policy strategies [38]. Few studies based on short-term observations in China have developed effective behavioral intervention strategies against smoking, physical inactivity, obesity, and hypertension-related NCDs [39]. However, current health system limitations in China restrict the success of such interventions, thereby justifying environmental and policy interventions, such as urban planning and design (to facilitate active living) and agricultural policies (to promote healthy diets and lifestyles) [39,40].

Environmental and public health studies in China have focused on the effects of environmental pollution (e.g., haze weather) on health, whilst the urbanization effects receive inadequate attention [39]. The effects of environments (e.g., social or built environments) on NCDs have been largely ignored [41] because the upsurge of NCDs in China is comparatively recent and so far there have been limited responses to it. Thus, this frontier research field has failed to attract sufficient attention and funding. For example, the two National Plans for NCD prevention and treatment recognize NCDs as “a critical public health problem in China”. However, both plans emphasize public health policy perspectives, such as behavioral/lifestyle interventions rather than environmental interventions [42]. Accordingly, neither plan explicitly refers to research on the environment and on NCDs [2,8].

As a result, research proposals that focus on the environmental approach to NCDs have received limited support from the Ministry of Science and Technology of China (MSTC) or the National Natural Science Foundation of China (NSFC), which are the two leading national funding bodies for basic research in China [41]. The MSTC continues to prefer biomedical approaches and technologies, as well as a focus on (end) treatment. In the MSTC’s latest application guide for 2017 key specialized projects on the prevention and control of significant NCDs, NCD prevention and control technologies still dominate the agenda. Meanwhile, environmental approaches have received minimal attention [43]. Unlike the socio-ecological approaches, these technologies focus on intrinsic factors, diagnoses, and the treatment of NCDs (as health outcomes) at the individual level, rather than focus on population-level interventions and (source) prevention. Likewise, the NSFC provides minimal funding to this line of research. Such prioritization in funding may negatively affect the effective prevention of NCDs in China in the long run.

Despite the strong emphasis on NCD prevention, the two National Plans recruit more common strategies or individual lifestyle interventions from clinical medicine, public health, or epidemiology (e.g., early diagnoses and treatment, screening, dietary change, and health education) [2,8]. Meanwhile, research on environmental factor and NCDs receives minimal attention and funding. With regard to the nearly 300 million Chinese individuals suffering from NCDs [2], the prevalent situation seriously impedes research and innovation. This impediment may render Chinese NCD prevention strategies administrative rather than scientific [41], wherein actions are progressing faster than the science.

## 4. Promoting China’s Research on the Environment and NCDs

Research on environmental factors and NCDs is still in its infancy stage, and it is concentrated in developed countries. Environmental effects on NCDs may vary across countries, such as China, that are experiencing rapid socioeconomic transitions and environmental changes. Moreover, empirical evidence on effective interventions across countries remains insufficient [1,9]. The distinct characteristics of China require innovative thinking and approaches to NCD research and prevention. For example, rapid urbanization, compact urban form, and exorbitant land prices have resulted in insufficient public facilities for physical activities in urban China. Furthermore, serious air pollution discourages physical activities in most Chinese cities. Therefore, China urgently needs to strengthen its research capacity building in this regard, as well as promote creative and robust research with the goal of formulating evidence-based interventions and effective prevention strategies against NCDs.

Public health is a priority in China’s national development strategy. At the highest level national health conference held on August 19, 2016, President Xi Jinping highlighted that the health of the people must underpin all policy making [44]. Apart from urbanization and aging, the environment is a key health factor. This resulted in the inclusion of health-supportive environments into the Medium- and Long-term Plans for the Prevention and Treatment of Chronic Diseases [8]. The enhanced political priority and emphasis would create sizable opportunities to enhance research on the environment and NCDs.

**Figure 1:**
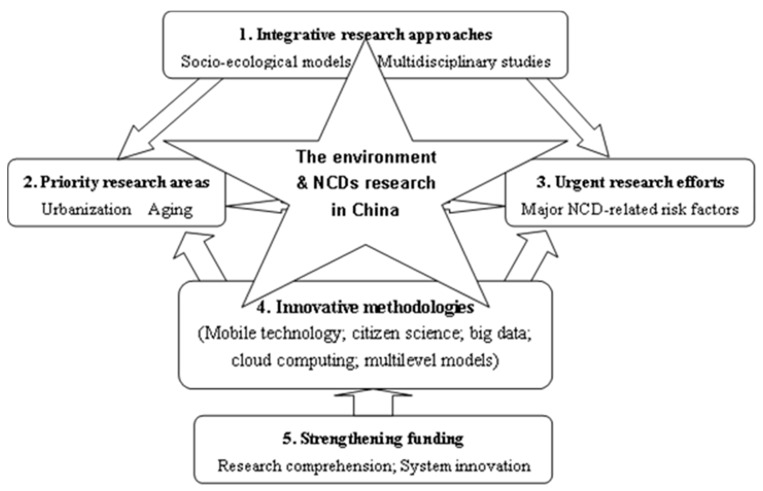
Comprehensive research perspectives on the environment and non-communicable chronic diseases (NCDs) in China.

In summary, this line of research should be promoted in China. However, several key aspects must be emphasized (Figure 1).

First, application of integrative (socio-ecological) approaches to NCDs is important, apart from the specific and separate focus on biomedical approaches or biotechnologies. Beyond environmental pollution, environments should be regarded as “multilevel, multidimensional, and multi-time-scaled” [14] (p.S65). Multilevel refers to the contextual dynamics at usually nested levels of analysis beyond the individuals, such as schools, workplaces, and neighborhoods. This broadly defined environment offers socially and geographically meaningful boundaries upon which the implementation of the public health programs can be facilitated [14]. Multidimensional covers not only the chemical or polluted environment, but also the various domains of peoples’ daily lives, such as social, economic, physical or built, institutional, and food environments. Studies have indicated that the NCD mortality rate is strongly associated with several social, environmental, economic, and behavioral factors [34]. This definition represents a comprehensive and integrative understanding of environments and it clarifies their relationships with reference to NCDs. The multi-time-scaled aspect adapted from life course theory incorporates the longitudinal or multi-temporal changes on individuals and within populations. This may facilitate the identification of specific environmental characteristics that engender certain NCDs or the causal links between them [36].

Integrative approaches require strong multidisciplinary collaborations. The task of disentangling complex multilevel, multidimensional, and longitudinal causal pathways that link the environment and NCDs cannot be attained without using a multidisciplinary approach. Multidisciplinary research may formulate integrated theories and inform complex interventions that typically target multiple outcomes simultaneously and that work across levels, dimensions, and timescales [45].

Second, urbanization (or urban environments) and aging should be prioritized. In urban China, rising urbanization and the associated lifestyles have significantly promoted the prevalence of major NCDs, such as cardiovascular disease, diabetes, cancer, and mental illnesses [3,39,46]. Moreover, the incidences of overweight and obesity amongst children in cities have significantly increased due to socioeconomic development [3]. As Yang et al. (2018) recently indicated in the Tsinghua–Lancet Commission on healthy cities in China, “Cities hold the key (role) to a healthy China” [46] (p. 2144). Aging remains the principal force driving the NCDs epidemic in China [3,5]. By 2040, the Chinese population aged ≥65 years will constitute nearly one-fifth of the total population, and aging alone may double the increase in mortality caused by cardiovascular diseases from 2000 to 2040 [5]. However, continued urbanization and aging may elevate the NCDs epidemic in China over the subsequent decades. Thus, research must focus on effective prevention strategies against NCDs, the promotion of healthy aging, and the creation of healthy and livable cities in China. For example, it may include addressing how to provide public facilities for physical activities in a compact and populated city, using healthy urban planning and design.

Third, tobacco use, excessive drinking, physical inactivity, and unhealthy diet are the leading NCD-related behavioral risk factors in China [3], and they must be urgently addressed. Tobacco use is the most pressing problem encountered in the prevention of NCDs, for example, the high ratios of tobacco use versus the low awareness of the associated health risks [5]. In China, smokers currently number 300 million people, with 28.1% being people aged over 15 years, whilst the per capita yearly alcohol consumption amongst Chinese adults in 2012 is three liters, with 9.3% of the drinkers consuming alcohol at a harmful rate [3]. Moreover, dietary changes and reduced physical activity have significantly increased cases of overweight and obesity, which may induce multiple NCDs [6]. In the long run, these conditions may negatively influence socioeconomic development in China. However, the two National Plans for NCD Prevention and Treatment remain based on public health policy perspectives, with a focus on individual behavioral interventions. Understanding the links between environmental factors and the key NCD-related risk factors may provide new insights into possible effective interventions and the prevention of NCDs in China (for instance, the food environment and dietary behaviors, as well as the socio-cultural environment around tobacco use and drinking).

Fourth, the integrative, multilevel, multidimensional, multi-time-scaled, and thus multidisciplinary nature of the environmental factors determines the complexity of research on the environment factors and NCDs. This calls for innovative research methodologies, including data collection and analysis methods, as well as promoting technological advances. In this regard, mobile technology-based citizen science approaches (e.g., mobile phones and wearable sensors to capture environmental and health data), as well as big data and cloud computing [47,48,49] resources deserve more attention. Moreover, beyond considering traditional socioeconomic and geospatial data, current mobile and big data associated with NCDs typically include real-time, dynamic, and multi-scaled or longitudinal data, as well as multidimensional and multilevel contextual information. Such complex data present huge challenges in their analysis and use, which warrants the development of new analytical methods and models to support this research in addition to the current models (e.g., multilevel analysis).

Finally, funding agencies need to fully acknowledge this new research and recognize its necessity and urgency, leading to well-informed decisions. The strong political will regarding health may lead to increased funding towards this research. Given that environments have complex relationships with NCDs, there is a need for prioritized funding to support integrated and multidisciplinary or interdisciplinary approaches rather than towards biomedical approaches or technologies alone. However, this approach requires innovative funding mechanisms or significant changes in the current funding system, because multidisciplinary research is typically less funded [50]. The funding requires incorporation into national programs, particularly into the programs developed by the MSTC and NSFC. Other tailored funding plans formulated by governments at all levels or by the relevant agencies require implementation, for example, special funds and grants for exploratory research that encourage multidisciplinary collaborations. Such multichannel funding systems may greatly benefit research on environment factors and NCDs. However, the complexities and the multidisciplinary nature of this research necessitate long-term and sustained funding.

## 5. Conclusions

The global reach of NCDs poses significant challenges to all nations. Controlling and reducing NCDs is part of the global health and development agenda. It is urgent for NCD prevention policies and practices to move beyond traditional end treatment (via biomedical approaches and individual interventions) and instead embrace a more promising source-prevention solution (environmental interventions) guided by socio-ecological approaches. We strongly believe that the framework proposed in this paper can promote relevant research, being one of the many factors contributing to this paradigm change.

Future research will need to test the framework’s general applicability by customizing it to specific contexts. In anticipation, its application to China, a country heavily influenced by NCDs, may significantly improve China’s NCD prevention efforts. China’s efforts in this regard may offer new insights and approaches regarding the prevention of NCDs, and it can positively contribute to the improvement of global health and well-being.
